# Frequent Implication of Multistress-Tolerant *Campylobacter jejuni* in Human Infections

**DOI:** 10.3201/eid2406.171587

**Published:** 2018-06

**Authors:** Euna Oh, Linda Chui, Junghee Bae, Vincent Li, Angela Ma, Steven K. Mutschall, Eduardo N. Taboada, Lynn M. McMullen, Byeonghwa Jeon

**Affiliations:** University of Alberta School of Public Health, Edmonton, Alberta, Canada (E. Oh, J. Bae, B. Jeon);; Provincial Laboratory for Public Health in Alberta, Canada, Edmonton (L. Chui, V. Li);; University of Alberta, Edmonton (L. Chui, A. Ma, L.M. McMullen);; Public Health Agency of Canada, Lethbridge, Alberta, Canada (S.K. Mutschall, E.N. Taboada)

**Keywords:** Campylobacter jejuni, bacteria, multistress tolerant, pathogen survival, transmission, campylobacteriosis, human infections, aerobic stress, disinfectant exposure, freeze-thaw, heat treatment, osmotic stress, Edmonton, Alberta, Canada

## Abstract

*Campylobacter jejuni*, a major cause of bacterial foodborne illnesses, is considered highly susceptible to environmental stresses. In this study, we extensively investigated the stress tolerance of 121 clinical strains of *C. jejuni* against 5 stress conditions (aerobic stress, disinfectant exposure, freeze-thaw, heat treatment, and osmotic stress) that this pathogenic bacterium might encounter during foodborne transmission to humans. In contrast to our current perception about high stress sensitivity of *C. jejuni*, a number of clinical strains of *C. jejuni* were highly tolerant to multiple stresses. We performed population genetics analysis by using comparative genomic fingerprinting and showed that multistress-tolerant strains of *C. jejuni* constituted distinct clades. The comparative genomic fingerprinting subtypes belonging to multistress-tolerant clades were more frequently implicated in human infections than those in stress-sensitive clades. We identified unique stress-tolerant *C. jejuni* clones and showed the role of stress tolerance in human campylobacteriosis.

*Campylobacter* spp., particularly *Campylobacter jejuni*, are a leading bacterial cause of gastroenteritis and cause ≈166 million cases of infection worldwide per year (*1*). Human exposure to *C. jejuni* occurs through various routes, including foodborne and waterborne transmission and direct contact with farm and companion animals (*2*). However, foodborne transmission accounts for most cases of human campylobacteriosis, mainly through consumption of contaminated poultry (*1*,*3*). *C. jejuni* inhabits chicken intestines as a commensal microorganism at a level >10^6^–10^8^ CFU/g of feces (*4*). Thus, release of fecal contents from chicken might contaminate poultry carcasses at multiple steps during poultry processing to finished product (*5*). To reduce *C. jejuni* contamination in poultry meat, various mitigation strategies are used in poultry processing, including chemical treatment with decontamination agents (*6*), physical treatment with hot water and steam, and chilling and freezing of carcasses (*7*).

Although *C. jejuni* is transmitted to humans through the food chain or by other routes in the environment, *C. jejuni* encounters a wide range of stress conditions before human exposure (Technical Appendix Figure). Stress tolerance plays a major role in transmission of foodborne pathogens to humans by enhancing bacterial survival during food processing, preservation, and cooking (*8*). For example, the capability of *Salmonella* spp. to survive in low-moisture environments enables this pathogen to contaminate dry foods (*9*), and the psychrotrophic nature of *Listeria monocytogenes* enables it to survive in ice cream (*10*).

Unlike these robust enteric pathogens, *C. jejuni* is considered highly susceptible to stresses primarily because of the lack of many stress response factors (*11*). Because of its stress sensitivity, *C. jejuni* is considered unlikely to survive effectively outside animal hosts. However, regarding food safety, it is still an enigma how this stress-sensitive bacterium survives under hostile conditions in various transmission routes and is increasingly responsible for human illnesses worldwide. Despite the role of stress tolerance during transmission of pathogens to humans, the role of stress tolerance in human infections has not yet been elucidated for *C. jejuni*. To fill this major knowledge gap, in this study, we extensively examined stress tolerance in 121 clinical strains of *C. jejuni* and detected stress-tolerant *C. jejuni* populations that are frequently involved in human infections.

## Materials and Methods

### Strains and Culture Conditions

Clinical strains of *C. jejuni* were provided by the Provincial Laboratory for Public Health in Alberta. These strains were obtained from 5 health zones (North, Edmonton, Central, Calgary, and South) that cover the entire province of Alberta. A total of 121 selected clinical strains of *C. jejuni* from human stool samples were tested: 24 from the North Zone, 48 from the Edmonton Zone, 13 from the Central Zone, 15 from the Calgary Zone, 11 from the South Zone, and 10 from unknown locations. We routinely grew strains in Mueller-Hinton medium at 42°C microaerobically (5% O_2_, 10% CO_2_, and 85% N_2_).

### Aerotolerance Test

The aerotolerance test was performed according to a method described in our previous study (*12*). We grew *C. jejuni* strains on Mueller-Hinton agar at 42°C overnight under microaerobic conditions. Strains were harvested, placed in fresh Mueller-Hinton broth, and diluted to an optical density at 600 nm (OD_600_) of 0.1. We incubated bacterial suspensions at 42°C aerobically with shaking at 200 rpm. Aliquots were taken at 0, 12, and 24 h for serial dilution and enumeration.

### Aerobic Survival of Strains on Refrigerated Raw Chicken

Survival of *C. jejuni* was determined as described (*13*). We prepared pieces of raw chicken skin (≈0.2 g/piece) from chicken thigh by cutting with a sterile razor and placed them into 96-well microtiter plates. The *C. jejuni* suspension prepared from overnight cultures was diluted to an OD_600_ of 0.07 in phosphate-buffered saline, and 100 μL of the *C. jejuni* suspension was applied to the chicken skin. The chicken skin pieces spiked with *C. jejuni* were stored at 4°C. We placed sterile needles under both sides of the lid of a microtiter plate to prevent blockage of air circulation. We placed a container filled with water near the plate to prevent samples from being dried during incubation. After incubation, chicken skin pieces were transferred to 15-mL tubes containing 1 mL of fresh Mueller-Hinton broth. After we vigorously vortexed samples for 2 min, we collected supernatants to enumerate bacteria by using Preston *Campylobacter*-selective agar.

### Stress Tolerance Tests

We grew the 121 clinical strains of *C. jejuni* on Mueller-Hinton agar overnight at 42°C under microaerobic conditions and resuspended them in Mueller-Hinton broth for stress tolerance tests as follows. Each assay was performed with negative controls without artificial contamination of *C. jejuni* and was repeated 3 times.

#### Resistance to Peracetic Acid, a Chemical Decontaminant

This experiment was performed on the basis of a previous study with slight modifications (*14*). We prepared a piece of raw chicken (≈0.2 g/piece) containing skin and muscle by using a sterile razor. We spiked each chicken piece with ≈10^8^ CFU of *C. jejuni* and incubated each piece at 4°C for 1 h under microaerobic conditions. The chicken piece was dipped in 750 ppm peracetic acid (PAA) (Sigma Aldrich, St. Louis, MO, USA) for 15 s and transferred into a 15-mL tube containing 1 mL fresh Mueller-Hinton broth. After we vortexed supernatants for 2 min, we serially diluted and spread them on Preston *Campylobacter*-selective agar to enumerate *C. jejuni*.

#### Tolerance to Freeze-Thaw

We diluted *C. jejuni* suspensions to an OD_600_ of 0.1 (≈10^8^ CFU/mL). Aliquots (≈100 µL) were applied to 0.2 g of chicken skin and placed in 96-well microtiter plates. After incubation at −20°C, samples were defrosted at 4°C for 2 h and transferred to a 15-mL tube containing 1 mL of Mueller-Hinton broth. After we vortexed supernatants for 2 min, we serially diluted them in Mueller-Hinton broth for enumeration by plating on Preston *Campylobacter*-selective agar.

#### Thermotolerance Test

The *C. jejuni* suspension from overnight cultures was 100-fold diluted in whole milk (3.25% milk fat). We transferred milk contaminated with *C. jejuni* to 96-well microtiter plates and subjected them to heat treatment by using a thermocycler (Eppendorf, Hamburg, Germany) at 72°C for 15 s or 30 s. After a serial dilution, samples were plated on Preston *Campylobacter*-selective agar.

#### Osmotolerance Test

After a 10-fold serial dilution, 5 μL of *C. jejuni* suspension was spotted on Mueller-Hinton agar (negative control) or Mueller-Hinton agar plates supplemented with 2% and 4% NaCl. We measured viability after overnight incubation at 42°C under microaerobic conditions.

### Comparative Genomic Fingerprinting Analysis

We performed the comparative genomic fingerprinting 40 (CGF40) assay as described (*15*) and stored data in a secured database at the Provincial Laboratory for Public Health in Alberta. We performed cluster analysis by using BioNumerics software version 7.6 (Applied Maths NV, Sint-Martens-Latem, Belgium) with the unweighted pair group method with arithmetic mean clustering algorithm and the simple matching coefficient.

### Multilocus Sequence Typing

We performed multilocus sequence typing (MLST) analysis of *C. jejuni* strains as described (*16*). We conducted PCR amplification of 7 housekeeping genes (*aspA*, *glnA*, *gltA*, *glyA*, *pgm*, *tkt*, and *uncA*) by using ExTaq polymerase (Takara, Tokyo, Japan). PCR products were sequenced by Macrogen Inc. (Seoul, South Korea), and sequences were analyzed by using the pubMLST database (https://pubmlst.org/).

### Statistical Analysis

We performed statistical analysis for aerotolerance and stress tolerance tests by using 2-way analysis of variance. We calculated Pearson correlation coefficients on the basis of correlations between level of stress tolerance and prevalence of *C. jejuni* in the CGF collection of human clinical isolates in Alberta. Statistical analyses were performed by using SPSS version 21 (IBM, Armonk, NY, USA).

## Results

### Prevalence of Hyperaerotolerant Strains in Human Clinical Cases

**Figure 1:**
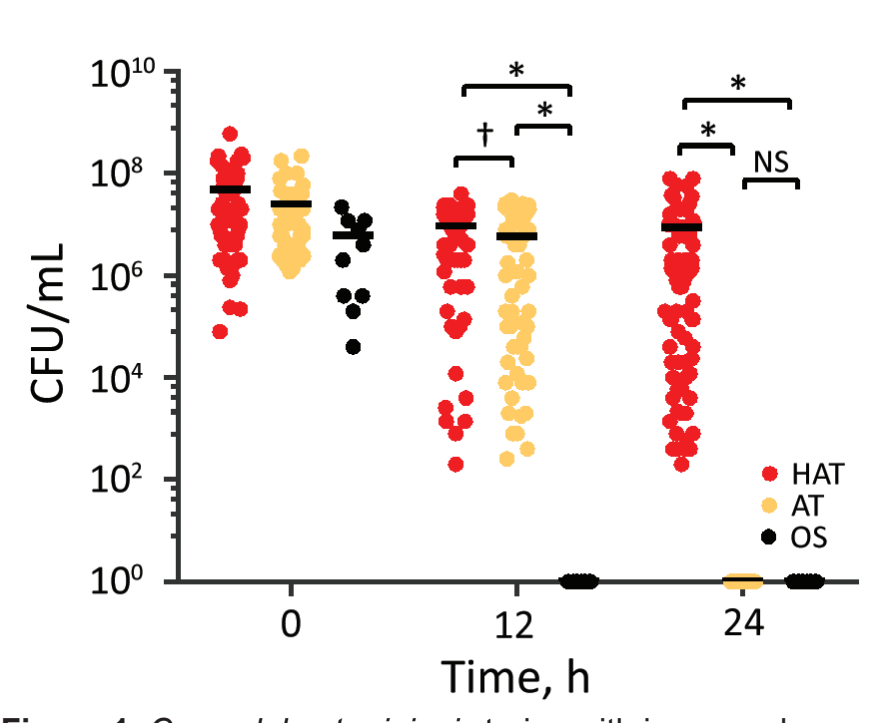
*Campylobacter jejuni* strains with increased aerotolerance in human clinical cases of campylobacteriosis. Results show levels of aerotolerance in 121 *C. jejuni* strains, including HAT (n = 65), AT (n = 46), and OS strains (n = 10). Clinical strains of *C. jejuni* were mostly AT and HAT. Results are representative of 3 independent experiments, and similar results were observed in all repeated experiments. Solid horizontal lines indicate average CFU. AT, aerotolerant; HAT, hyperaerotolerant; NS, not significant; OS, oxygen sensitive. *p<0.0001; †p<0.001.

Because aerotolerance plays a major role in survival of *C. jejuni* under aerobic conditions, we determined the aerotolerance of the 121 clinical strains. Strains that did not survive aerobic shaking at 200 rpm for 12 h were considered oxygen sensitive; those that survived for ≈12–24 h were classified as aerotolerant, and those that survived for >24 h were classified as hyperaerotolerant (Figure 1). Most clinical strains of *C. jejuni* were hyperaerotolerant (65/121, 53.7%) and aerotolerant (46/121, 38.0%); only 8.3% (10/121) of strains were oxygen sensitive (Figure 1). Results showed that hyperaerotolerant and aerotolerant strains of *C. jejuni* are highly prevalent in cases of human infection.

### Survival of Hyperaerotolerant Strains in Refrigerated Chickens under Aerobic Conditions

**Figure 2:**
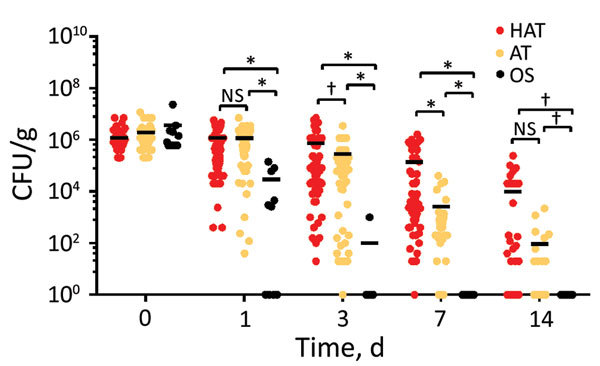
Extended survival of HAT *Campylobacter jejuni* strains on refrigerated raw chicken under aerobic conditions. Results show CFU of 121 *C. jejuni* strains, including HAT (n = 65), AT (n = 46), and OS (n = 10). Results are representative of 3 independent experiments, and similar results were observed in repeated experiments. Solid horizontal lines indicate average CFU. AT, aerotolerant; HAT, hyperaerotolerant; NS, not significant; OS, oxygen sensitive. *p<0.0001; †p<0.05.

We determined the viability of the 121 clinical strains of *C. jejuni* on refrigerated raw chicken. When we compared oxygen-sensitive *C. jejuni* strains with aerotolerant and hyperaerotolerant strains, the aerotolerant and hyperaerotolerant strains survived for longer periods on chicken at refrigeration temperatures in the air. Both aerotolerant and hyperaerotolerant strains were recovered after 7 days; however, only 1 oxygen-sensitive strain was recovered after 3 days (Figure 2). Some hyperaerotolerant strains survived on poultry meat after 2 weeks under aerobic conditions with only a marginal reduction in CFU (<1 log CFU/g meat) (Figure 2). Results showed that aerotolerant and hyperaerotolerant strains survived on refrigerated chicken longer than oxygen-sensitive strains.

### Prevalence of Clinical Strains Tolerant to Environmental Stresses

#### Disinfectant Exposure

**Figure 3:**
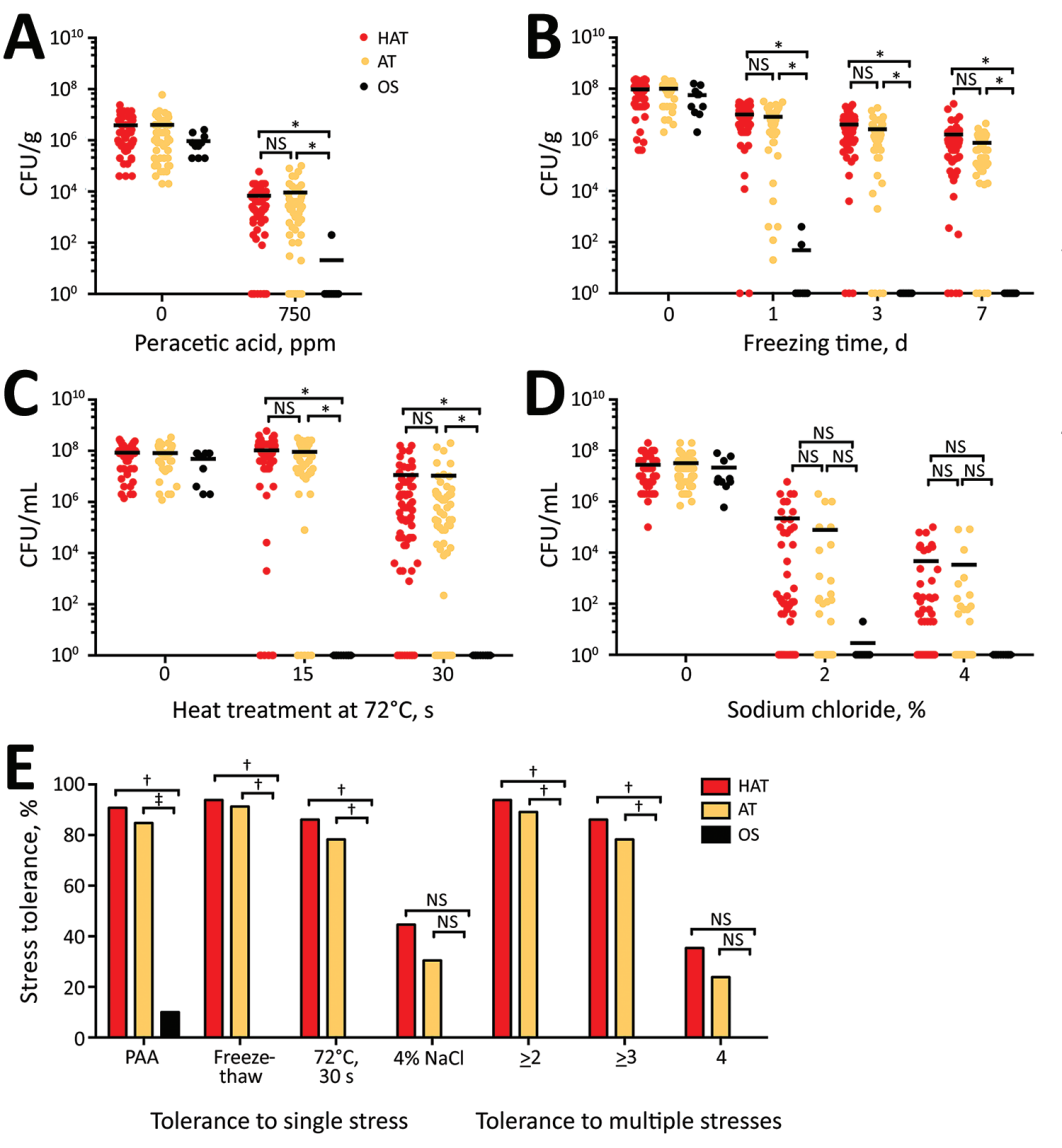
Tolerance to multiple stresses in 121 clinical strains of *Camplyobacter jejuni*. A) Resistance of *C. jejuni* to disinfectant (PAA) on chicken. B) Freeze-thaw tolerance of human clinical strains of *C. jejuni* on chicken. C) Tolerance to heat treatment of clinical *C. jejuni* strains in milk. D) Osmotolerance of clinical strains of *C. jejuni*. E) Percentage of stress-tolerant strains in each aerotolerance group: HAT (n = 65), AT (n = 46), and OS (n = 10). Results are representative of 3 independent experiments, and similar results were observed in all repeated experiments. Solid horizontal lines indicate average CFU. AT, aerotolerant; HAT, hyperaerotolerant; NS, not significant; OS, oxygen sensitive. *p<0.00001; †p<0.01; ‡p<0.05.

Decontamination with antimicrobial agents is a common harsh stressor that *C. jejuni* encounters on poultry carcasses during processing. Among the decontamination agents that are used during poultry processing (*6*), PAA is highly effective in reducing *C. jejuni* load on poultry carcasses and is widely used (*14*). PAA is a mixture of acetic acid and hydrogen peroxide, and spontaneously decomposes to acetic acid, oxygen, and water (*17*). Most hyperaerotolerant (59/65, 90.8%) and aerotolerant (39/46, 84.8%) strains survived exposure to PAA; however, only 1 oxygen-sensitive strain survived with a major reduction in CFU (Figure 3, panels A, E). Results show that aerotolerant and hyperaerotolerant *C. jejuni* strains are highly tolerant to PAA.

#### Freezing

Because freezing has been reported to decrease the prevalence of *Campylobacter* spp. in chicken, freezing is considered a major measure in controlling *C. jejuni* contamination in poultry products (*18*,*19*). We inoculated the 121 clinical strains onto raw chicken pieces and subjected the pieces spiked with *C. jejuni* to freezing at −20°C. After 3 days of freezing, none of the oxygen-sensitive strains survived. However, 93.8% (61/65) of the hyperaerotolerant strains and 91.3% (42/46) of the aerotolerant strains survived, even after 7 days of freezing (Figure 3, panels B, E), which demonstrated that hyperaerotolerant and aerotolerant strains were highly tolerant to freezing.

#### Heat Treatment

Heat treatment during pasteurization and cooking processes might kill pathogenic bacteria in foods. Although *C. jejuni* is a thermophile and grows optimally at 42°C, it is more sensitive to heat treatments than other enteropathogenic bacteria (*11*). We examined heat tolerance by exposing *C. jejuni* in milk to high-temperature short-time (HTST) pasteurization conditions (72°C for 15 s). A total of 83.5% (101/121) of clinical strains were tolerant to HTST pasteurization conditions, and 76.0% (92/121) of strains survived even after an extended heat treatment (72°C for 30 s) (Figure 3, panel C). Although no oxygen-sensitive strains survived HTST pasteurization conditions, 86.2% (56/65) of hyperaerotolerant strains and 78.3% (36/46) of aerotolerant strains survived after heat treatment at 72°C for 30 s (Figure 3, panels C, E). Results showed that the level of thermotolerance was also different depending on the strain.

#### Osmotic Stress

Compared with other enteric pathogens, *C. jejuni* is considered highly sensitive to osmotic stress and easily inactivated by >2% NaCl (*20*). However, 44.6% (54/121) of the 121 clinical strains survived 2% NaCl, and 35.5% (43/121) of the strains were tolerant, even in 4% NaCl (Figure 3, panels D, E; Technical Appendix Table). In contrast to our knowledge about *C. jejuni*, most clinical strains of *C. jejuni* were highly tolerant to high salt concentrations.

### Identification of Clades Tolerant to Multiple Stresses

**Figure 4:**
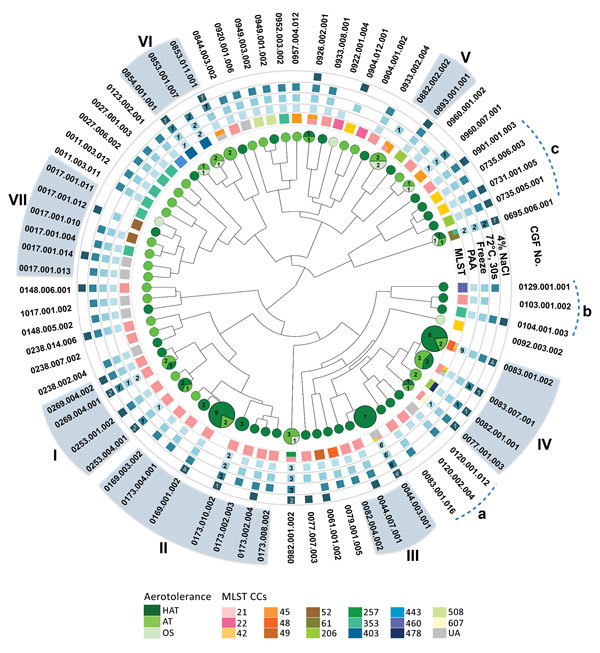
Clonal distribution of MST *Campylobacter jejuni* strains from human clinical cases of campylobacteriosis. The phylogenetic tree was generated from CGF40 profiles. Circles indicate aerotolerant strains, and squares indicate for stress-tolerant strains. A square without a number indicates all tested strains in the subtype were stress tolerant. Clades I–VII were identified on the basis of the analysis criteria (90% similarity cutoff and >5 strains/clade). Additional clades that consisted predominantly of HAT strains, but did not meet the criteria, were named a, b, and c. AT, aerotolerant; CCs, clonal complexes; CGF, comparative genomic fingerprinting; HAT, hyperaerotolerant; MLST, multilocus sequence typing; OS, oxygen sensitive; PAA, peracetic acid; UA, unassigned.

On the basis of the high prevalence of *C. jejuni* in human clinical isolates tolerant to multiple stresses, we hypothesized that the multistress-tolerant (MST) strains are more likely to cause human illnesses because they can overcome various stresses in food processing, preservation, and cooking. This feature might enable MST *C. jejuni* strains to establish unique clones by outcompeting stress-sensitive *C. jejuni*. To examine this hypothesis, we conducted a population genetics analysis by using the CGF40 method, which is based on allelic assessment of ≈40 different genes in *C. jejuni* (*15*). Using a 90% similarity cutoff, we found that 7 clades containing >5 strains were identified in the CGF40 dendrogram (Figure 4). Three additional clades were also found to be highly populated with hyperaerotolerant strains (clades a, b, and c) (Figure 4), but these strains were excluded from further analysis because they did not meet the analysis criteria (i.e., 90% cutoff and >5 strains/clade). Each clade consisted of strains belonging to different MLST clonal complexes (CCs). For example, all strains in clades I and II belonged to MLST CC 21, whereas strains in clade IV belonged to several different MLST CCs (Figure 4). Nevertheless, hyperaerotolerant *C. jejuni* strains clustered in the CGF40 dendrogram (Figure 4), and hyperaerotolerant *C. jejuni* strains were found predominantly in clades I–IV (Figure 4). These results suggest that hyperaerotolerance might be associated with unique genetic backgrounds in *C. jejuni*.

### Association of Stress Tolerance with Human Infections

**Figure 5:**
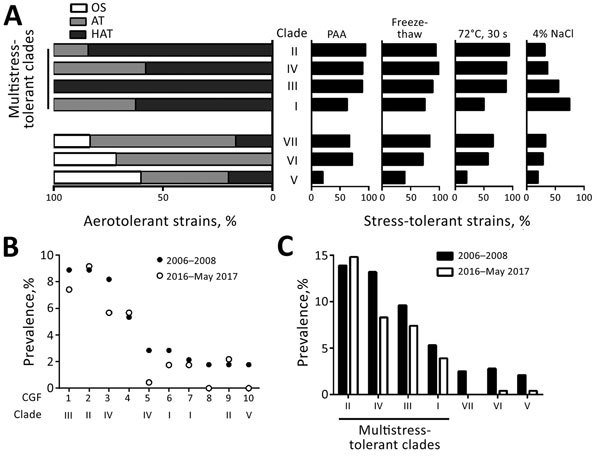
Increased stress tolerance and prevalence of multistress-tolerant (MST) clades of *Camplyobacter jejuni*. A) Augmented tolerance of MST clades to 5 stressors (aeration, disinfectant [PAA], freeze-thaw, heat, and salt). Clades are arranged in the order of decreasing levels of overall stress tolerance. B) Top 10 most prevalent CGF subtypes in the CGF collection of human clinical strains of *C. jejuni* in Alberta, Canada, during 2006–2008 (n = 281) and 2016–May 2017 (n = 229). 1, 0044.003.001; 2, 0169.001.002; 3, 0083.001.002; 4, 0695.006.001; 5, 0083.007.001; 6, 0269.004.001; 7, 0253.004.001; 8. 0123.002.001; 9, 0173.004.001; 10, 0882.002.002. C) Cumulative prevalence of the CGF subtypes belonging to MST clades in the CGF collection of human clinical strains of *C. jejuni* in Alberta, Canada, during 2006–2008 (n = 281) and 2016–May 2017 (n = 229). AT, aerotolerant; CGF, comparative genomic fingerprinting; HAT, hyperaerotolerant; OS, oxygen sensitive; PAA, peracetic acid.

We analyzed stress tolerance levels of the strains in each clade. Strains in clades II, III, and IV were highly tolerant to PAA, freeze-thaw, and heat treatment (Figure 5, panel A). When we compared strains in clades II, III, and IV with strains in clade I, strains in clade I were relatively less tolerant to PAA, freeze-thaw, and heat treatment, but highly tolerant to osmotic stress (Figure 5, panel A). Clades V, VI, and VII, which included mostly oxygen-sensitive and aerotolerant strains, showed relatively reduced stress tolerance in comparison with clades I–IV. Strains in clade V were highly sensitive to all stresses tested (Figure 5, panel A). Each clade showed differential levels of tolerance to different stresses. Because of high levels of tolerance to multiple stresses for clades I–IV, we now call them MST clades.

To investigate effects of stress tolerance on strains that cause human campylobacteriosis, we examined how frequently CGF subtypes of MST clades were involved in cases of human infection. The 3 most frequent CGF subtypes (0169.001.002, 0044.003.001, and 0083.001.002) in the CGF collection of human clinical *C. jejuni* strains in Alberta isolated during 2006–2008 belonged to MST clades (Figure 5, panel B). In addition, these 3 CGF subtypes showed the highest prevalence in the recent (2016–May 2017) collection of *C. jejuni* clinical strains in Alberta (Figure 5, panel B). The total prevalence of all CGF subtypes in each clade indicates that MST clades have been a persistent cause of human campylobacteriosis in Alberta (Figure 5, panel C). Furthermore, the prevalence of *C. jejuni* in human clinical cases showed a statistical correlation with level of hyperaerotolerance (Pearson correlation coefficient [r] = 0.797; p<0.05), disinfectant (i.e., PAA) resistance (r = 0.771; p<0.05), freeze-thaw tolerance (r = 0.773; p<0.05), and heat tolerance (r = 0.825; p<0.05), whereas the level of osmotolerance was not associated with prevalence (r = 0.18; p = 0.699). These results strongly suggest that stress tolerance of *C. jejuni* plays a critical role in development of human infections, and that the MST clades include the primary *C. jejuni* strains causing human campylobacteriosis.

## Discussion

*C. jejuni* is considered a fastidious and highly stress-sensitive bacterium. In contrast to this perception, most clinical strains of *C. jejuni* tested in this study showed increased tolerance to multiple stresses. MST *C. jejuni* clones were identified in this study by combining extensive stress tolerance testing with a population genetics analysis (Figure 4; Figure 5, panel A). Furthermore, we demonstrated that MST *C. jejuni* clones are the primary cause of human campylobacteriosis in Alberta (Figure 5, panels B, C). The findings in this study strongly suggest that stress tolerance in *C. jejuni* is a critical determinant affecting human campylobacteriosis. These findings would help to answer the unresolved issue about how this fastidious, stress-sensitive bacterial pathogen is increasingly responsible for an increase in human illnesses worldwide; some *C. jejuni* strains are highly stress tolerant.

On the basis of findings in this study, we can speculate that MST *C. jejuni* strains can overcome and survive in hostile stress conditions in food processing and various environmental niches during transmission to humans more effectively than stress-sensitive strains. Consequently, MST *C. jejuni* strains are more likely to be transmitted to humans than stress-sensitive strains because these strains might be enriched and form unique clones by repeating the cycle of environmental survival, transmission, and human infection. The predominant CGF subtypes in MST clades (0169.001.002, 0044.003.001, and 0083.001.002) are not only prevalent in the CGF collection of human clinical *C. jejuni* strains in Alberta but also in the Canadian *Campylobacter* CGF database, which contains CGF information for >25,000 *C. jejuni* strains from foods, animals, humans, and environmental samples across Canada (*21*). Presumably, the level of stress tolerance for clinical strains will be different from that for nonclinical strains because clinical strains have already undergone a range of stress conditions during transmission and infection. We are currently investigating stress tolerance in nonclinical strains of *C. jejuni* from foods.

Strains in CGF subtype 0044.003.001 in MST clade III were highly tolerant to multiple stresses, including PAA, freezing, and heat treatment (Figure 4). The CGF subtype 0044.003.001 was commonly found in human clinical cases in Alberta (Figure 5, panel B) and Canada (*21*). This CGF subtype is detected more frequently in human clinical cases (59.0%) than in animals (40.5%) in the Canadian *Campylobacter* CGF database (*21*). According to a recent study from Nova Scotia, a province geographically distant from Alberta, the predominant CGF subtype 0083.001.002 in MST clade IV is also the most common CGF subtype found in human clinical isolates of *C. jejuni* in this province (*22*). CGF subtype 0083.001.002 is primarily associated with chicken (*22*), and strains in 0083.001.002 were highly tolerant to PAA, freezing, and heat treatment (Figure 4). This finding suggests that multistress tolerance may facilitate *C. jejuni* survival in poultry processing, preservation, and cooking, and consequently might increase chances of food contamination and human exposure.

Because of the common understanding about high-stress sensitivity in *C. jejuni*, food contamination by *C. jejuni* has been relatively underestimated when compared with that for other robust foodborne pathogens. For other enteric pathogens, high-risk strains (e.g., *Escherichia coli* O157:H7) are strictly monitored and controlled during food inspection. However, such risk-based strain differentiation is not performed for *C. jejuni*, and current food safety policies regarding *Campylobacter* spp. are based on total *Campylobacter* spp. count in foods. The findings in this study suggest that the MST *C. jejuni* clones might be the primary target to monitor and control to address the major public health issue of *Campylobacter* infections.

Most of the stress conditions tested in this study, such as freezing, refrigeration, heat treatment, and high salt concentrations, are commonly used in poultry processing and cooking in many countries. Thus, findings of this study might not be limited only to strains of *C. jejuni* from Canada. However, tolerance to antimicrobial disinfectants in *C. jejuni* might vary because each jurisdiction has a different policy on use of disinfectants and this might affect the possibility of *C. jejuni* exposure to specific disinfecting substances. For example, PAA is used widely to decontaminate poultry carcasses in Canada and the United States. However, the European Union recently performed safety and efficacy analysis of PAA application on poultry carcasses (*23*). Given the well-known genetic diversity in *C. jejuni*, MST *C. jejuni* clones might have different genotypes in different geographic locations. Future studies should aim to compare multistress tolerance in historical and recent strains of *C. jejuni* from different countries. In addition, further investigations are required to elucidate the molecular mechanisms of multistress tolerance in *C. jejuni*.

Technical Appendix Additional information on human infections with multistress-tolerant *Campylobacter jejuni*.
