# Supplementary material for: Frequent Implication of Multistress-Tolerant Campylobacter jejuni in Human Infections
Source: Emerg Infect Dis. 2018 Jun;24(6):1037–44. doi: 10.3201/eid2406.171587 (PMC6004869; doi:10.3201/eid2406.171587)
Supplement: Technical Appendix — Additional information on human infections with multistress-tolerant Campylobacter jejuni. [file 17-1587-Techapp-s1.pdf]

Article DOI: https://doi.org/10.3201/eid2406.171587

Frequent Implication of Multistress-Tolerant *Campylobacter jejuni* in Human Infections

Technical Appendix

Technical Appendix Table. Stress tolerance of clinical strains of *Campylobacter jejuni**

Characteristic	PAA	Freeze-thaw	Heat, 72°C		NaCl		No. stresses		
			15 s	30 s	2%	4%	>2	>3	4
HAT, n = 65	59 (90.8)	61 (93.8)	60 (92.3)	56 (86.2)	35 953.8)	29 (44.6)	61 (93.8)	56 (86.2)	23 (35.4)
AT, n = 46	39 (84.8)	42 (91.3)	41 (89.1)	36 (78.3)	17 (37.0)	14 (30.4)	41 (89.1)	36 (78.3)	11 (23.9)
OS, n = 10	1 (10.0)	0 (0.0)	0 (0.0)	0 (0.0)	2 (20.0)	0 (0.0)	0 (0.0)	0 (0.0)	0 (0.0)

*Values are no. (%). Percentages were calculated on the basis of the total number of stains in each aerotolerance group. Strains tolerant to 72°C for 30 s were considered heat tolerant, and those tolerant to 4% NaCl were considered osmotolerant. AT, aerotolerant; HAT, hyperaerotolerant; OS, oxygen sensitive; PAA, peracetic acid.

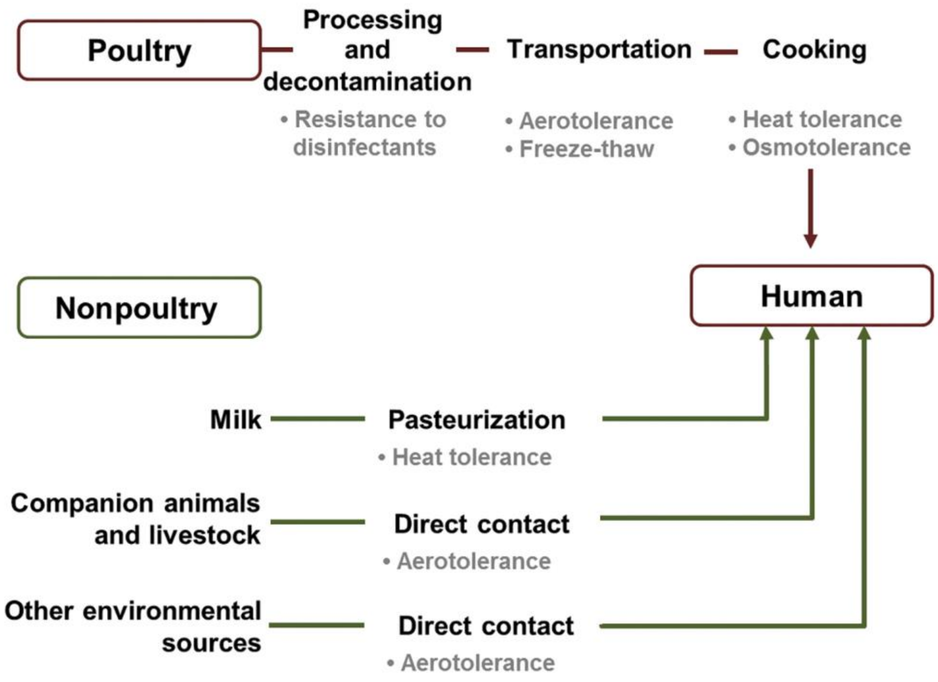

Technical Appendix Figure. Stress conditions encountered by *Camplylobacter jejuni* in the food chain or environment before human exposure.
